# Management of Osteomyelitis-Induced Massive Tibial Bone Defect by Monolateral External Fixator Combined with Antibiotics-Impregnated Calcium Sulphate: A Retrospective Study

**DOI:** 10.1155/2018/9070216

**Published:** 2018-12-19

**Authors:** Chenghe Qin, Lei Xu, Juan Liao, Jia Fang, Yanjun Hu

**Affiliations:** ^1^Department of Orthopedics and Traumatology, Guangdong Second Provincial General Hospital, 466 Xingang Road C., Haizhu District, Guangzhou 510317, China; ^2^Department of Orthopedics and Traumatology, Li Ka Shing Faculty of Medicine, the University of Hong Kong, Hong Kong SAR 999077, China; ^3^Department of Pediatric Intensive Care Unit, Guangzhou Women and Children's Medical Center, Guangzhou Medical University, 9 Junsui Road, Zhujiang New Town, Tianhe District, Guangzhou 510623, China; ^4^Department of Orthopedics and Traumatology, Nanfang Hospital, Southern Medical University, 1833 Guangzhou North Avenue, Baiyun District, Guangzhou 510515, China

## Abstract

**Aim:**

The present study is aimed at evaluating the effect of combined treatment on massive bone defect using radical debridement, antibiotic calcium sulphate, and monolateral external fixator.

**Methods:**

35 patients with tibial osteomyelitis received radical debridement, and during surgery antibiotics-impregnated calcium sulphate was used for filling the bone defect. Monolateral external fixator was used to manage the bone defect of average 95 (61-185) cm.

**Results:**

Bone union was achieved in 34 patients (97.1%) with no reinfection. One case was presented with reinfection and further debridement was conducted. The average time for the utility of external fixation was 17 (7-32) months, and external fixation index (EFI) was 1.79 mon/cm. The mean follow-up duration after surgery was 33.7 (21-41) months. 19, 13, and 3 patients got excellent, good, and fair bone results, respectively. Meanwhile, functional results were excellent, good, fair, and poor in 13, 15, 6, and 1 patient. The most common complication was pain (100%) and superficial pin-tract infection (22.8%). Delayed maturation was incurred in 2 patients.

**Conclusion:**

Our study reveals that radical debridement combined with antibiotics-impregnated calcium sulphate can suppress infection, and distraction osteogenesis using monolateral external fixators plays an effective role in managing osteomyelitis-induced massive tibial bone defect.

## 1. Introduction

Osteomyelitis is a concept of inflammation induced by pyogenic bacteria in bone and bone marrow following trauma or surgical procedures. Due to the increasing use of orthopedic implants for treatment of fracture, more and more cases of osteomyelitis are presented. For example, of more than 2 million fixation devices consumed annually in United States alone, on average 5% of implanted devices become infected and for each case more than US$ 15000 are estimated for treatment [1].

Characterized by the presence of sequestrum, chronic osteomyelitis is usually developed due to the delayed and poor treatment of acute osteomyelitis. It is difficult to treat because its management usually relies on integrated approach. Age, smoking, alcohol abuse, and lack of nutritious support all served as risk factors. Even standard procedure is followed; therapeutic failure or infectious recurrence still ranges from 20% to 30% [2, 3].

A successful debridement is of great importance to cure osteomyelitis, and this usually indicates a considerable bone and soft tissue defect. Because infection is easily spread to a large area, the resultant dead space is necessary to be eliminated by filling with either surrounding muscle or flap. In recent decades, an increasing number of surgeons prefer to use antibiotics-impregnated carriers to fill the debridement-induced dead space, since it has many advantages compared with other methods [4].

Distraction osteogenesis has been largely used in the reconstruction of lower limbs because of its limited invasiveness and versatility. However, to the best of our knowledge, previously only one study [5] was found to report the treatment of infected long bone massive defect (>6 cm) by distraction osteogenesis combined with antibiotics-impregnated carrier. The carrier used in their treatment was PMMA, which was nonabsorbable and therefore additional surgeries for removal were needed. In contrast, we used the absorbable calcium sulphate as carrier. Therefore, in the present study we describe our successful experience in the management of osteomyelitis-induced massive tibial defect.

## 2. Including Criteria and Patients' Information

Between January 2008 and July 2013, 98 consecutive patients who had infected tibial bone defect were treated surgically in our institute. The patients including criteria were (1) patients ≥18 and ≤60 years old; (2) bone defect was initially caused by treatment of osteomyelitis; (3) the osteomyelitis is graded as type of 4A/B according to the Cierny-Mader grading system [6]; (4) bone defect was more than 6cm; (5) antibiotics-impregnated calcium sulphate was embedded immediately after radical debridement; and (6) bone defect was eventually managed by distraction osteogenesis. On the contrary, bone defects due to excision of bone tumors and congenital defect, and simple aseptic nonunion were excluded from the study. Accordingly, 35 patients were included in our study. Our study was approved by the ethics committee of the institute and the informed consent was taken from all the patients.

**Figure 1:**
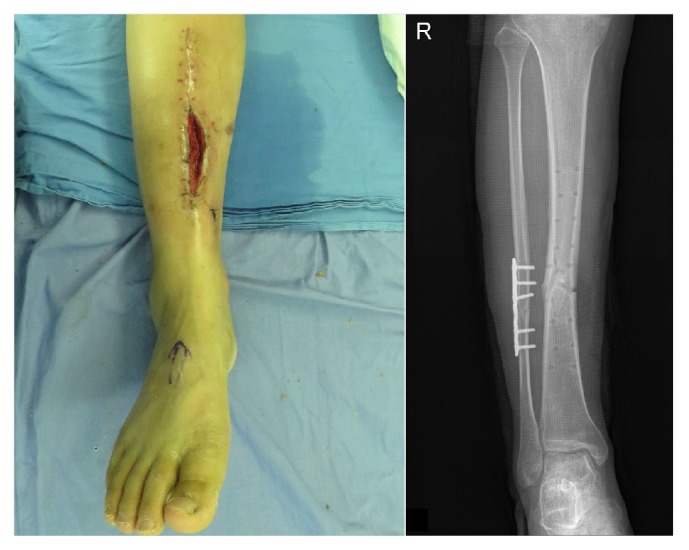
Case 17, a 33-year-old man with chronic osteomyelitis of the tibia. The left figure is the preoperative clinical photograph. The right figure is the preoperative anteroposterior X-ray photograph.

Our study had 26 (74.3%) males and 9 (25.7%) females with an average age of 38 years old. The initial diagnosis was closed fracture in 11 patients and open fracture in 24 patients (Table 1), and the latter was further classified by Gustilo and Anderson grading system [7]. Most of the patients underwent several operations in various medical institutes, including debridement and open reduction and internal fixation (ORIF). Road traffic accident, falling from the height, and machine crush injury were the most common causes of initial fracture (Figure 1).

At admission to our institute each patient routinely received physical examination and laboratory tests. Occasionally they presented with vague symptoms including pain, atrophic nonunion, pain, draining sinus, swelling, local warmth, erythema at involved site, and necrosis of wound edges. However, the results of laboratory tests revealed that inflammatory markers such as erythrocyte sedimentation rate (ESR), the C-reactive protein (CRP), and white blood cell count (WBC) did not change correspondingly. Moreover, all patients received plain radiographs and computed tomography (CT) tests. Bone resorption, sequestration, periosteal or endosteal new bone formation, cortical irregularities, and atrophic nonunion were found in most cases.

## 3. Surgical Procedures

**Figure 2:**
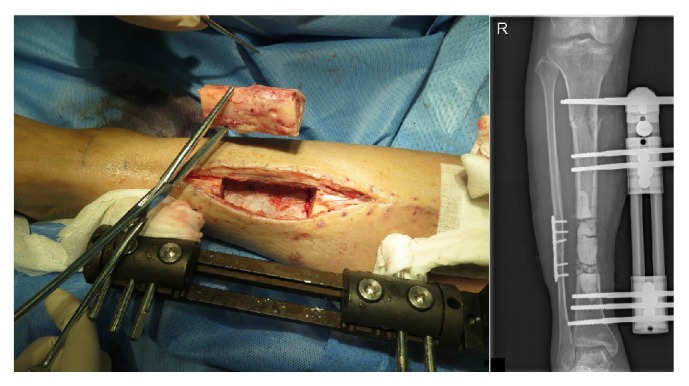
The left figure was the resection of infected bone segment when radical debridement was conducted. The right figure was the anteroposterior X-ray photograph taken few hours after surgery, and antibiotic-impregnated calcium sulphate was demonstrated to be filled in the bone defect.

The patients were positioned supine on a radiolucent operating table, and the incision was performed along the previous surgical incision. With the assistance of C-arm fluoroscopy, infected internal implants were taken out. With regard to the cases used to have internal intramedullary nail, the canal was reamed and irrigated after nail removal. Subsequently, the infected bones and soft tissues were adequately exposed by elongated cut, and thorough debridement was conducted. Specifically, infected nonunion, the necrotic bones, and devitalized soft tissues were totally debrided (Figure 2). During debridement, the necrotic tissues obtained at least at 3 different representative spots were collected for aerobic and anaerobic culture to identify the responsible pathogenic bacteria. Bone specimens and purulent secretion were also collected and sent for histological examination for confirmation of osteomyelitis and exclusion of malignant changes caused by chronic inflammation process. The debridement continued until the viable cortical bone and raw bleeding were observed [8], and the infected scarred tissues and discharging sinus tracts were sufficiently eradicated.

The thorough debridement was followed by corticotomy or acute compression. In our study, there were 27 patients whose fibulas were intact or used to have internal fixation, and acute compression could not be performed. Other 8 patients' fibular had fracture, defect, or infection which needed to be managed, and therefore acute compression was conducted. The acute compression was incomplete, which left 3-5 cm defect behind. During compression, the peripheral blood circulation in distal extremity should be paid attention not to be disturbed. Afterwards, the wound was irrigated by copious amount of saline, and the gloves of all surgeons as well as surgical tools were changed. The debridement-induced bone void as well as the medullary cavity was filled with antibiotics-impregnated calcium sulphate (Stimulan®, Biocomposite Ltd., UK) (Figure 2). Before surgery, each patient received antibiotic sensitivity test which determined the antibiotics (vancomycin and/or gentamycin) used for impregnation. Notably, they should not be used in the patients who presented with hepatic failure or renal failure, which in our study was not found in any cases. Normally 0.5g vancomycin was mixed with 10cc calcium sulphate, while 160K international unit gentamycin was mixed with 10cc calcium sulphate. It is noteworthy that the antibiotic spacer was shaped in consistent with the original shape of resected bone, and more was filled in the medullary cavities of both bony stumps (Figure 5). Afterwards, primary wound closure with drainage tubes was performed in patients with adequate coverage of soft tissues. Otherwise delayed wound closure was performed after the wound was temporally covered by vacuum sealing drainage for 3 days at most, when clean granulated tissue was formed without purulent secretion.

## 4. Postoperative Treatment

Systemic antibiotics were performed immediately after surgery. Specifically, initially the intravenous broad-spectrum antibiotics were used empirically before the results of sensitivity came out (usually less than 72 hours) and were subsequently modified according to the culture and sensitivity results. After use for two weeks, it was replaced by oral antibiotics according to the sensitivity results. At the same time, CRP, ESR, and routine blood test were regularly monitored. The antibiotic treatment was continued for a minimum of 6 weeks or until the ESR and CRP had returned to normal level [9].

Regular changes of sterile wound dressing were conducted. Effusion seeping out of the wound, which normally demonstrated as white, foamy fluid, was also checked. Once wound dressing was soaked by such effusion, it was changed immediately.

**Figure 3:**
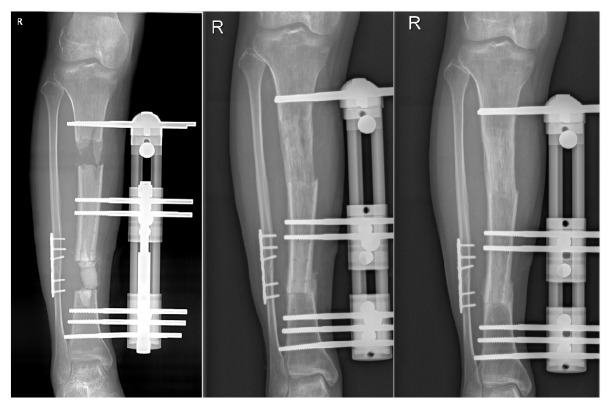
The figures in the left, middle, and right position showed the anteroposterior X-ray photographs taken about 1, 8, and 13 months after surgery.

All patients were encouraged to perform partial weight-bearing exercises such as walking with clutches as early as the third or fourth day after the surgery. Distraction was started after a latency period of 7 to 14 days at a rate of 0.25mm three or four times a day (Figure 3). The distraction rate was modified according to the regeneration quality evaluated by radiographs. Radiographs were taken regularly every 2 and 4 weeks at the outpatient department visit during the distraction period and the consolidation period, respectively. At the same time, the physical examinations were performed for the evaluation of the limb function, and the detection and treatment of the concomitant complications were also carried on. The external fixator was removed when the radiographs showed the appearance of consolidated bridging callus and the limb length was restored.

## 5. Outcome Evaluation

The functional assessment of all the patients was performed according to the criteria proposed by Paley et al. [10], which includes bone results and functional results. Bone results have four items for evaluation (bone union, infection, deformity, and limb length discrepancy), and the functional results have five items for evaluation (observable limp, adjacent joint stiffness, dystrophy of soft tissue, pain, and incapability of motivation such as unemployment or inability to return to daily activity). The outcome of both parts can be categorized into four grades such as excellent, good, fair, and poor.

## 6. Results

According to the measurement by radiographic analysis system after the surgery, the average length of bone defect was 9.5 cm (6 to 18.5 cm). The culture results of the samples obtained from the surgery can be found in Table 2. All patients were persisted to the follow-up and the mean follow-up period was 33.7 months (range from 25 to 41 months). The laboratory results and radiographs were available for all the patients.

**Figure 4:**
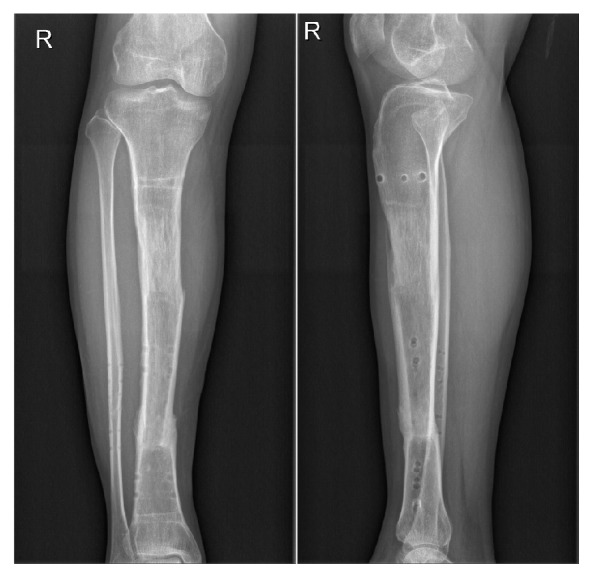
Anteroposterior and lateral X-ray photographs taken 3 months after the external fixation was removed.

**Figure 5:**
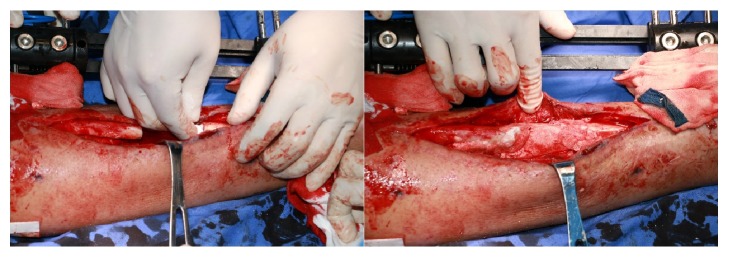
Techniques to place antibiotics-impregnated calcium sulphate. Please note that both bone marrow cavity (the left figure) and bone defect (the right figure) should be filled.

Successful bone union was achieved in 34 patients without any reinfections (Figure 4). The average time for external fixation was about 17 months (range from 7 to 32 months). The mean external fixation index was the application duration of external fixation in months divided by the total length of bone transport in centimeters, which was 1.79 mon/cm (range from 1.13 to 2.79 mon/cm) in our study. According to the Paley's grading system, the details of bone results and functional results for each patient were shown in Table 2.

The treatment in one patient (case 7) was regarded as “failure,” because this case had reinfection and thus repeated debridement was needed. The patient also received anterolateral thigh perforator flap for considerable soft tissue defect. Although bone union was achieved, but foot drop was presented.

## 7. Complications

Pain was the most common complaint, in particular when distraction period was started. It was relieved after oral analgesics were received. 8 patients presented with tract infection (case 7, 12, 15, 19, 22, 26, 30, and 32) which was managed by local tract care and oral and/or intravenous administration of broad-spectrum antibiotics. Delayed maturation occured in 2 patients (case 24 and 28) and additional surgeries were done by implanting graft harvested from the patients' iliac bones under general anesthesia between the docking ends of bone segments. No compartment syndrome was found in our study.

Three patients (case 10, 20, 14) had knee stiffness and were partially relieved after arthrolysis and physical therapy. Secondary osteotomy was conducted in case 27 because of wrong adjustment during bone lengthening, which resulted in early new bone consolidation. It was followed by ankle fusion due to the fact that the distal end of tibia was lost in debridement.

## 8. Discussion

As revealed in our study, the osteomyelitis in most patients usually resulted from infected fracture and/or surgical interventions. The infection is the cause of delayed bone union or nonunion, and causes bone defect. Therefore, complete cure of infection is the mainstay of treatment. However, the chronic osteomyelitis is so obstinate that even normal debridement and consistent systemic administration of antibiotics can hardly achieve a successful cure. Although the concept of “burning an infection in the fire of an Ilizarov technique” was described by Ilizarov himself [11], it has altered to that only radical debridement until live and bleeding bone is reached can suppress infection [6, 12].

At the admittance to our institute, the laboratory test revealed that the serum inflammatory markers such as CRP, ESR, and WBC were elevated only in some of the patients. For example, only 14 (37.1%), 25 (71.4%), and 22 (62.8%) patients had elevated level of WBC count, ESR, and CRP, respectively, at admittance to our institute. And such changes seem not to be related to the local signs of inflammation. This is thought to be partially attributed to the fact that inflammatory process in some patients was too weak to trigger CRP and/or ESR production [13]. For example, it was revealed that about 15% of bone infection participants and 60% of soft tissue infection participants were reported to have negative CRP level [14]. It may also be explained by the kinetics of inflammatory markers, because CRP has a half-life of 18 hours, starts rising within 4-6 hours and peaks in 48 hours, and finally returns to normal within 3-7 days [15]. Likewise, ESR takes few weeks to return normal [16]. 6 weeks after surgery, ESR and CRP returned to normal level in 17 (68%) and 19 (86.4%) cases, respectively. This may indicate that chronical osteomyelitis is a quiescent infection in many cases, and the inflammatory markers alone have not sufficient sensibility, and laboratory tests should be combined with clinical manifestations, radiological tests, whereas the negative test cannot exclude the infection [9, 17]. On the other hand, only persistently increased ESR and CRP implied active bone infection [14], which was not found after treatment in our study and indicated that our combined treatment approach was effective.

The radical debridement is essential to cure infection, but this can lead to a considerable dead space. Since it is usually quickly filled with hematoma and provides an ideal culture medium for bacteria [18], it may lead to recurrence of infection. In our study, antibiotics-loaded spacer is used for filling the inflexible tissue space and releases antibiotics slowly and constantly, maintaining the local antibiotic concentration to a satisfactory level for weeks, avoiding the potential side effect of constant systemic antibiotic use [4, 19]. Furthermore, it has structural similarity to natural bone [20] and is osteoconductive, which potentially promote the regeneration of bone. Although it was reported to be accompanied with prolonged wound ooze [21], it was self-limiting and had no significant influence on reinfection rate.

Calcium sulphate, however, has some limits. For example, in our study some patients had complications of pain during bone transport. This might be related to the blocking effect of calcium sulphate on bones during transportation. Normally calcium sulphate is still presented in the radiographs 7 to 14 days after surgery; therefore, it is assumed that its interaction with bone could stimulate the peripheral soft tissues and cause pain to patients. However, in our experience such blocking effect would have no influence on the overall treatment. After all, it is the antibiotics-impregnated calcium sulphate that plays the critical role in suppressing infection and creates a prerequisite for the following bone transport.

The combined approaches of management could effectively reduce the need for docking site bone graft. For example, only two cases in our study needed additional docking site bone graft. This is supposed to be related to satisfactory suppression of infection due to radical debridement and effectiveness of systemic and local antibiotics. It is also assumed that the embedment of antibiotics-impregnated calcium sulphate (osteoconductivity) and soft tissue defect could reduce the needed revision of scar at docking site.

Bone reconstruction is also very important in the whole treatment of osteomyelitis. Although free fibular transplantation and Masquelet technique are applicable, however, these techniques usually require multiple surgical procedures and could hardly achieve reliable consolidation and weight-bearing function during treatment. Amputation is an alternative treatment and its hospital cost is obviously less than that of bone transport, but its long-term cost is thought to be higher if the lifetime costs of prostheses are included [22, 23]. In comparison, distraction osteogenesis can regenerate living bone with same length, width, and strength as that of the native bone and restore the injured soft tissues to the proportion to the lengthening bone [24]. Besides, with the increasing reusing of apparatus exterior to limb, the cost of external fixator is considerably reduced and therefore affordable.

The principal concerns arising from the massive bone defect involve poor stability and soft tissue envelop. It is known that increased size of defect results in terrible mechanical quality and worse histological resuscitation. Therefore, we preferred to use monolateral external fixation because its impact on the soft tissue is limited compared with conventional internal fixation. Moreover, the stable and flexible fixation provided by monolateral external fixation not only achieve greater mechanical stability and callus formation [25], but also reconstruct the limb alignment and reduce the risk of pin and screw loosing [26]. Although circular external fixation is also commonly used for managing bone defect, we focused on monolateral external fixation because it is much easier for surgeons to learn and can be rapidly applied with minimal equipment, and its relatively lower price and less cumbersome burden can be better accepted by patients [27].

The average external fixation index (EFI) in our study was 1.79 (1.13 to 2.79) mon/cm. Compared with the previous studies, Harshwal et al. [28] reported an EFI of 1.44 mon/cm for a mean defect of 5.7cm using monofocal treatment and 2.5 mon/cm for a 1.9 cm mean defect using bifocal treatment, Megas et al. [29] reported an EFI of 1.06 mon/cm for a mean defect size of 5.0 cm using a combined external fixation following an intramedullary nail, and CW Oh et al. reported an EFI of 0.44 mom/cm for a mean defect size of 5.9 using a combined external fixation with locking plate technique [30]. Such discrepancy may be attributed to different treatment approaches, diverse mean bone defect size, and the presence of infection or not. More comparisons between effect of our approach and other techniques under uniform standards are needed. It is speculated that age and complications could have a close relationship with EFI, whereas the influence caused by the previous operation number and the type of infecting organism seem to be limited.

## 9. Conclusion

Our study suggests that the combined treatment plays an effective role in managing osteomyelitis-induced massive tibial bone defect. Radical debridement is important and antibiotics-impregnated calcium sulphate effectively suppresses infection. The technique in our study is easy to learn and can effectively correct both bone defect and deformity.

## Figures and Tables

**Table 1:** Clinical descriptions of the 35 patients in our study.

Case No.	Age (years)	Sex	Initial injury mode	Classification of fracture	No. of previous operation	Abnormal serum inflammatory markers*∗*
1	42	M	TA	Open/Gustilo II	2	WBC, ESR,
2	33	F	TA	Closed	2	CRP
3	20	M	TA	Open/ Gustilo I	2	CRP
4	45	M	TA	Closed	3	ESR, CRP
5	40	M	FH	Closed	1	WBC, ESR
6	23	M	CIM	Open/ Gustilo I	4	ESR, CRP
7	52	M	TA	Open/ Gustilo III	2	WBC, ESR
8	41	M	FH	Closed	4	WBC, CRP
9	26	F	FH	Open/ Gustilo II	5	ESR
10	54	M	TA	Open/ Gustilo I	3	ESR, CRP
11	31	M	TA	Open/ Gustilo I	3	WBC, ESR
12	43	M	CIM	Open/ Gustilo II	1	WBC, ESR
13	54	F	TA	Open/ Gustilo II	1	ESR, CRP
14	55	F	TA	Closed	2	ESR, CRP
15	57	M	TA	Closed	3	CRP
16	29	M	CIM	Open/ Gustilo II	2	ESR, CRP
17	33	M	TA	Open/ Gustilo I	2	CRP
18	18	M	TA	Open/ Gustilo I	3	ESR, CRP
19	27	M	FH	Open/ Gustilo III	2	CRP
20	27	F	TA	Open/ Gustilo I	2	WBC, ESR, CRP
21	60	F	CIM	Open/ Gustilo II	1	ESR
22	49	M	TA	Closed	2	ESR,
23	35	M	CIM	Open/ Gustilo II	2	ESR, CRP
24	34	M	TA	Closed	1	WBC, ESR
25	53	M	TA	Closed	1	ESR, CRP
26	20	M	TA	Open/ Gustilo II	3	ESR
27	58	F	TA	Closed	2	WBC, CRP
28	19	M	TA	Open/ Gustilo III	2	WBC, CRP
29	45	M	CIM	Closed	2	ESR, CRP
30	54	M	FH	Open/ Gustilo III	2	WBC, CRP
31	45	F	TA	Open/ Gustilo II	1	ESR, CRP
32	18	M	TA	Open/ Gustilo II	2	WBC, CRP
33	18	M	TA	Open/ Gustilo III	1	WBC, ESR
34	24	F	TA	Open/ Gustilo I	2	ESR
35	50	M	FH	Open/ Gustilo II	1	ESR

TA: traffic accident; FH: fall from height; CIM: crush injury by machine.

*∗*Serum inflammatory markers revealed by the laboratory test at the admittance to the institute. WBC means the abnormal white blood cells level, ESR means the abnormal erythrocyte sedimentation rate level, and CRP means the abnormal C-reactive protein level.

**Table 2:** Detailed information about the treatment and outcome of the 35 patients.

Case number	Bone defect (mm)	Infection mechanism	EFT (mo.)	EFI (mo/cm)	Bone result	Functional result
1	139	Negative	22	1.58	Excellent	Excellent
2	66	SA	9	1.36	Excellent	Good
3	62	PA	7	1.13	Excellent	Excellent
4	75	EC	12	1.60	Good	Good
5	72	SA	12	1.67	Good	Good
6	120	AB	17	1.42	Excellent	Excellent
7	130	Negative	32	2.46	Fair	Poor
8	82	SA	14	1.71	Good	Excellent
9	87	SA	16	1.84	Excellent	Good
10	127	AB	20	1.57	Fair	Fair
11	83	Negative	14	1.69	Excellent	Good
12	171	EC	29	1.70	Good	Fair
13	103	SM	21	2.04	Good	Fair
14	64	SM	11	1.72	Good	Fair
15	67	SA	11	1.64	Excellent	Good
16	129	SA	17	1.32	Excellent	Excellent
17	63	SA	13	2.06	Good	Excellent
18	105	EC	16	1.52	Excellent	Excellent
19	111	SA	23	2.07	Good	Good
20	107	PA	19	1.78	Fair	Fair
21	104	EC	21	2.01	Excellent	Good
22	83	SA	14	1.69	Excellent	Excellent
23	93	SA	19	2.04	Good	Excellent
24	70	SA	18	2.57	Excellent	Excellent
25	80	EC	11	1.38	Excellent	Good
26	85	SA	13	1.53	Excellent	Excellent
27	68	PA	19	2.79	Good	Good
28	69	SA	18	2.63	Excellent	Good
29	75	SA	15	2.00	Excellent	Good
30	62	Negative	13	2.10	Good	Fair
31	85	AB	18	2.12	Excellent	Excellent
32	92	KP	17	1.85	Good	Good
33	185	KP	32	1.73	Good	Good
34	61	SA	11	1.80	Excellent	Good
35	152	Negative	29	1.91	Excellent	Excellent

SA: Staphylococcus Aureus; SM: Serratia Marcescens; AB: Acinetobacter Baumannii.

KP: Klebsiella Pneumoniae; PA: Pseudomonas Aeruginosa; EC: Escherichia Coli.

## Data Availability

The data used to support the findings of this study are available from the corresponding author upon request.
